# Pyorrhœa Alveolaris

**Published:** 1894-04

**Authors:** C. N. Peirce

**Affiliations:** Philadelphia


### PYORRHŒA ALVEOLARIS.[1]

BY C. N. PEIRCE, D.D.S., PHILADELPHIA.

In response to your request that I shall give you some thoughts upon "pyorrhœa alveolaris" for the monthly meeting of your District Dental Society, to be held on the 26th, in Buffalo, New York, I may say that it must be short, though I will try and make it to the point.

In writing, thinking, or speaking of the subject, "pyorrhœa alveolaris," the matter of first importance is to have it closely, definitely defined; without this limitation, there is no certainty that any two are dealing with the same pathological condition. Observation and interpretation, then, must enter largely into an intelligent comprehension of the subject, and in its discussion it must be borne in mind that we have different abnormal states, which are liable to be

[1] Read before the Eighth District Dental Society, Buffalo, N. Y., February 26, 1894.

spoken of as having a common origin, because of the similarity in results, this necessarily leading to antagonisms and embarrassment. Calcic pericementitis may have its origin at the gingival borders, the tartar acting as a local and mechanical irritant with the train of concomitant evils, such as irritation, inflammation, formation of pus, and absorption of gum and alveolar process. This may be treated locally, and be reported as a case of pyorrhœa cured, when the most that can be said of it is that it was the result of a local cause, and has been treated and cured by topical applications. Now, to avoid such complications in diagnosis, let us first recognize the fact that we have three distinct abnormal conditions affecting the gums, pericemental or alveolo-cemental membrane, and alveolar process. The first is gum inflammation and destruction caused by a mechanical irritant,—the salivary calculus of which we have just spoken. Second, inflammation of the gingival borders without the presence of tartar,—Dr. Black's phagedenic gingivitis and pericementitis. The third is a pericemental irritation commencing at or near the apical extremity of the root, due to the presence of some morbid composite of the blood, exuded with the plasma and infiltrating the alveolo-cemental membrane and frequently deposited or precipitated upon the root of the tooth near its apex. This latter I designated true pyorrhœa alveolaris or hæmatogenic pericementitis; and so intimately is it associated with some other local manifestation of a gouty diathesis that I have believed, and do now believe, it to be but another local expression of *that* systemic condition. This assumption has been confirmed by the fact that the accumulation which is so frequently found upon the roots of teeth near their apical extremity, by careful chemical analysis, has been proved to be composed of sodic urate, calcic urate, free uric-acid crystals, with some calcic phosphate and carbonate. Nor is this the only evidence of the correctness of this theory; cases which had been treated for months and indeed years without more than modifying the progress of the disease have, when the patient has been subjected to rigid constitutional treatment such as restriction in meat diet to simple albuminous food, with such remedies administered as are usually beneficial in gout or a uric-acid diathesis, responded promptly, the inflammatory state with its concomitants, pus formation and process absorption, has been arrested, and, if not too far progressed towards dislodgement, the teeth have become comparatively firm and free from pain.

I might in a few condensed propositions give conclusions which appear to me to be fully warranted:

1. The inflammatory stage of true pyorrhœa alveolaris primarily begins in tissues on the side of the root near the apical extremity, and, *secondarily*, advances towards the gingival borders.

2. The cause of this inflammation or pericementitis is the plasma exudation from the blood-vessels, freighted with salts, which, in their deposition and crystallization upon the cementum of the root, exert the influence of foreign bodies and react as irritants.

3. The salts in question as disclosed by chemical analysis, are calcium and sodium urates, free uric acid, and traces of calcium phosphate.

4. The chemical nature of these salts indicates a condition of the blood in which there is an excess of uratic salts and uric acid, due to either increased formation or imperfect elimination. The excess of these salts general pathologists regard as indicative of a faulty nutrition and the immediate cause of a series of local disturbances to which the term gouty has been applied, the nutritional disturbance being known as the uric-acid diathesis.

5. Results from constitutional, in connection with the usual local treatment in a number of well-authenticated cases of pyorrhœa have been so markedly satisfactory that the writer feels fully justified in his assumptions regarding the origin of the disease.

